# Virus-Induced Hypercoagulable State: A Mythical Cause for Cerebral Venous Sinus Thrombosis in an Adult Male

**DOI:** 10.7759/cureus.78118

**Published:** 2025-01-28

**Authors:** Nithish Nanda Palanisamy, Nandha Kumar Selvam, Sentamilselvan Vijayan, Vijayakumaran Ethiraju

**Affiliations:** 1 Internal Medicine, Coimbatore Medical College, Coimbatore, IND; 2 Emergency Medicine, Kovai Medical Center and Hospital, Coimbatore, IND; 3 Radiology, Kovai Medical Center and Hospital, Coimbatore, IND

**Keywords:** acquired protein s deficiency, cerebral venous sinus thrombosis (cvst), emergency medical service, mr venogram, varicella zoster virus infection

## Abstract

Chickenpox is a common childhood disease that presents with erythematous rashes. In adults, varicella can cause complications with significant morbidity. Cerebral venous sinus thrombosis (CVST) is a life-threatening condition if not promptly treated. Here, we report a rare case of CVST complicating primary varicella-zoster virus infection.

We present a case of a 34-year-old immunocompetent male with diffuse maculopapular rash and acute neurological deficit. Magnetic resonance venography revealed acute thrombosis involving the superior sagittal sinus, straight sinus, right transverse sinus, and confluence of sinuses. While arterial infarcts are well-known, venous thrombosis is less documented, particularly during active infection.

The underlying mechanism may involve a hypercoagulable state induced by the infection or direct viral invasion of the venous endothelium, leading to endothelial damage and subsequent thrombosis.

## Introduction

Varicella-zoster virus (VZV) is the etiologic agent of varicella (chickenpox), a childhood exanthematic disease that develops as a result of primary infection, and zoster (shingles), caused by reactivation of the virus persisting in a latent form in the dorsal sensory ganglia [1]. Although varicella is generally a mild self-limiting illness, it can have a serious clinical course in immunocompromised subjects and adults, which can lead to permanent damage to the central nervous system [1]. The incidence of VZV ranges from 13 to 16 cases per 1,000 persons per year, with substantial yearly variation. In tropical countries, acquisition of varicella occurs at a higher overall mean age with a higher proportion of cases in adults [2-4]. Diagnosis of varicella and zoster is most often made clinically on the basis of the characteristic generalized or unilateral dermatomal vesicular rashes, respectively [5]. Although arterial thrombosis is common in VZV, venous thrombosis is rare and may result from a similar mechanism as arterial thrombosis or acquired protein S deficiency, typically occurring after a latent period of two to three weeks, during which autoantibodies to natural anticoagulants develop, triggering widespread thrombosis.

This case report shows how prompt recognition and treatment of immunocompetent adult-complicated varicella can decrease mortality and progression of the infection.

## Case presentation

A 34-year-old male who had developed chickenpox presented to our emergency department in a drowsy state with complaints of right-sided limb weakness and speech disturbance for the past eight hours. The illness started with maculopapular rash predominantly on the trunk and limbs and to a lesser degree on the face eight days back. The lesions were centripetal and were diagnosed to be chickenpox. The lesions were in different stages when the patient developed neurological symptoms. He had symptoms of severe headache, nausea, and vomiting for the past one day. No other associated complaints of vision disturbance, seizure episodes, or loss of consciousness were noted. He was getting native treatment for chickenpox in his home. His medical history or surgical history was insignificant. He was not a smoker or alcoholic and had no history of drug abuse. His family history was also found to be insignificant.

**Figure 1:**
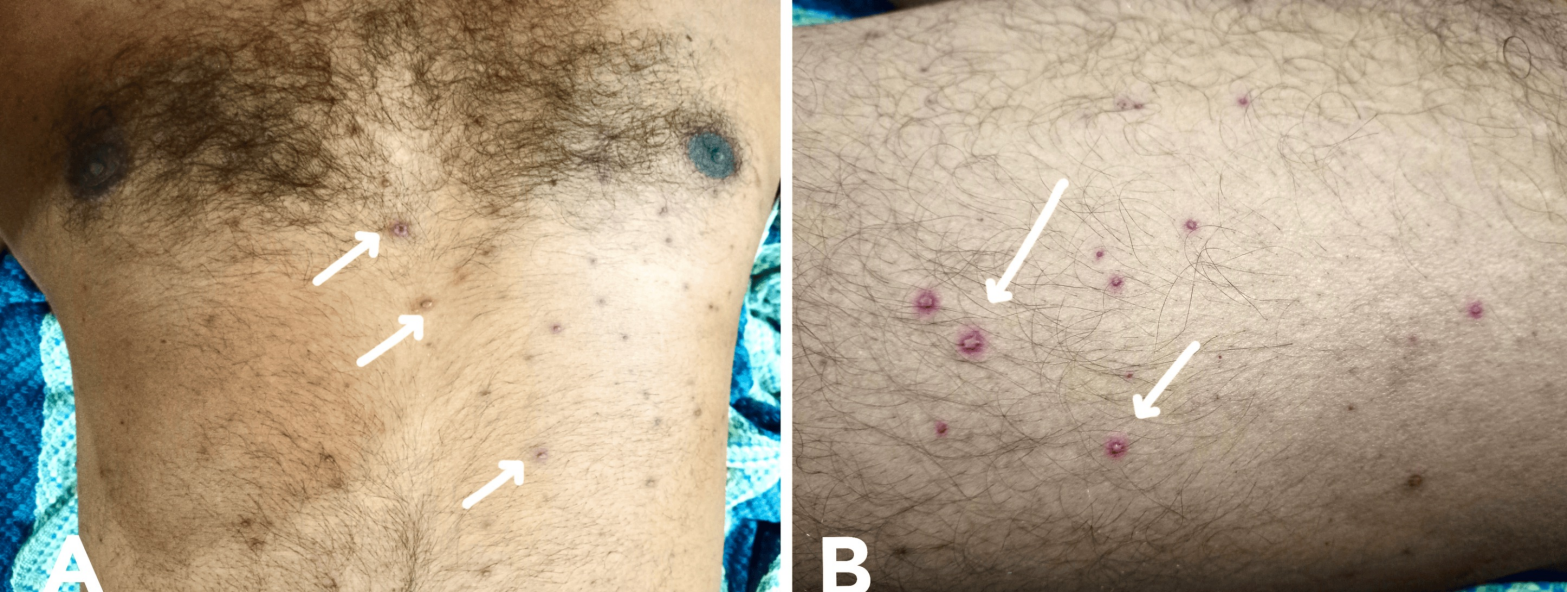
Diffuse maculopapular rash. Diffuse maculopapular rash with vesicles and pustules along with a few crusted lesions were present. The rash of chickenpox typically occurs in crops and is at different stages of evolution, as seen in this patient.

On general examination, the patient was drowsy, dehydrated, and afebrile. In the primary survey, his vital parameters were oxygen saturation (SpO2) of 96% on room air, respiratory rate (RR) of 18/minute, heart rate (HR) of 92 bpm, blood pressure (BP) of 110/70 mm Hg, random blood sugar (RBS) of 108 mg/dL, and temperature of 97.6°F, with a Glasgow Coma Scale (GCS) score of 10 (E3V1M6). Diffuse maculopapular rashes with lesions of different stages were present on the trunk and limbs (Figure 1).

Neurological examination revealed right-sided hemiparesis with right upper limb power of ⅖ and right lower limb power of ⅖, with motor aphasia. He was arousable to call and obeyed commands. Pupils were bilateral equal, reacting to light. Fundus was normal. A cranial nerve examination revealed no abnormalities. Sensory examination and cerebellar function tests cannot be completed due to his drowsy state. Other system examinations had no significant findings.

With a high clinical suspicion of cerebrovascular disease, an MRI of the brain with magnetic resonance venography (MRV) was ordered along with routine blood and biochemical investigations. Treatment was initiated with IV crystalloids (1 L bolus followed by maintenance), IV acyclovir 750 mg (stat followed by every eight hours (Q8H)), and other supportive measures. Ryle's tube and Foley's catheter were placed.

**Figure 2:**
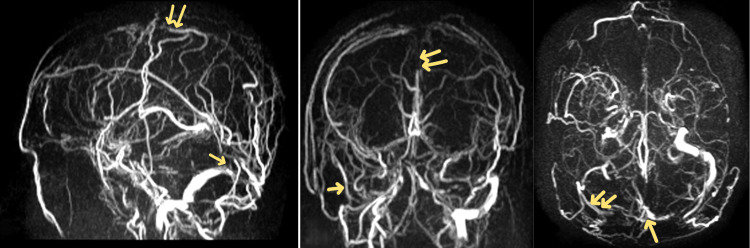
Magnetic resonance venography. Magnetic resonance venography revealed acute thrombosis of the superior sagittal sinus, straight sinus, right transverse sinus, and the confluence of sinuses.

ECG and chest X-ray were normal. MRV revealed acute thrombosis of the superior sagittal sinus, straight sinus, right transverse sinus, and the confluence of sinuses (Figure 2).

**Figure 3:**
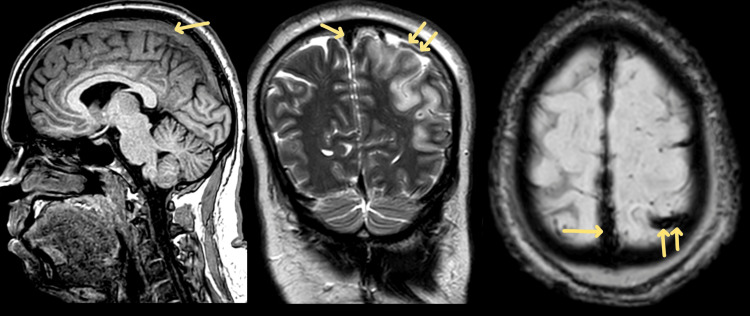
Magnetic resonance imaging T1/T2/susceptibility weighted imaging (SWI) sequences. MRI T1/T2/SWI sequences further suggested acute thrombosis of the superior sagittal sinus & cortical vein.

MRI T1/T2/susceptibility weighted imaging (SWI) sequences further suggested acute thrombosis of the superior sagittal sinus and cortical vein (Figure 3).

**Figure 4:**
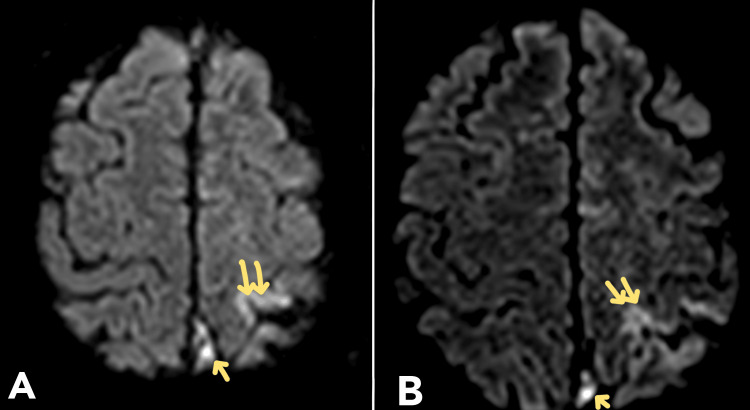
Magnetic resonance imaging diffusion-weighted imaging (MRI-DWI). MRI-DWI sequence revealed gyriform diffusion restriction noted in the periolandic region of the left high parietal lobe, likely to be ischemia.

MRI diffusion-weighted imaging (DWI) sequence revealed gyriform diffusion restriction noted in the periolandic region of the left high parietal lobe, likely to be ischemia (Figure 4).

His complete blood count, renal function test, liver function test, serum electrolytes, lipid profile, coagulation profile, vitamin B12, and serum homocysteine level were within normal limits. Hepatitis B surface antigen and HIV were non-reactive.

Considering the above MRI findings, the diagnosis of cerebrovascular venous sinus thrombosis complicating varicella infection was made, and the patient was started on low molecular weight heparin (LMWH; enoxaparin 40 mg s/c every 12 hours (Q12H)) along with diuretics (Glytol 75 ml IV Q8H) and a prophylactic antiepileptic drug (brivaracetam 50 mg IV Q12H) in the emergency medical resuscitation unit and transferred to neurology ICU. Additional thrombophilia workup of functional assays for antithrombin III, protein S deficiency, polymerase chain reaction (PCR) for factor V Leiden mutation, and testing for antiphospholipid antibodies and homocysteine levels revealed elevated antiphospholipid IgM antibody and deficient protein S (Table 1).

**Table 1:** Thrombophilia workup. Thrombophilia workup revealed elevated antiphospholipid IgM antibody and deficient protein S. Protein S is required for the degradation of coagulation factors Va and VIIIa along with protein C. Antiphospholipid antibodies are antibodies directed against phospholipid–protein complexes. These are hypercoagulable states associated with an increased risk for arterial or venous thrombosis, thrombocytopenia, and recurrent miscarriages [6]. ELISA: enzyme-linked immunosorbent assay.

Reported profiles	Results	Reference range
Antiphospholipid IgM antibody, serum (ELISA)	53.89	Normal = <12 U/mL; equivocal = 12-18 U/mL; positive = >18 U/mL
Antiphospholipid IgG antibody, serum (ELISA)	9.9	Normal = <12 U/mL; equivocal = 12-18 U/mL; positive = >18 U/mL
Protein C activity (clot-based assay)	103.0	67-195%
Protein S activity (clot-based assay)	36	77-143%

He was intubated on the second day due to deteriorating mental status and was started on IV benzodiazepine infusion and an antiepileptic agent (IV lacosamide 100 mg twice a day). On day four of the ICU stay, the patient tolerated the continuous positive airway pressure (CPAP) trial and was extubated. Post extubation, he was given adequate physiotherapy. He tolerated oral feeds and had normal bowel and bladder function.

After 12 days of hospital stay, he showed good clinical improvement with right-sided limb power improved to ⅘ and able to walk without support. Hence, he was discharged in stable condition and advised to continue oral antiepileptic and oral anticoagulant drugs till the next review, which was after two weeks.

## Discussion

This is a case of active varicella infection complicated by cerebral venous sinus thrombosis (CVST). The diagnosis of varicella was made clinically and neurological signs prompted brain imaging, which revealed CVST. The patient came to the emergency room with a GCS of 10 and required the need for intubation by day two due to worsening neurological symptoms.

Neurological complications of varicella in immunocompetent and immunosuppressed adults are associated with increased morbidity [7]. Varicella can cause arterial infarcts through direct invasion and cerebral arterial vasculopathy [8]. Although arterial thrombosis is common in VZV, venous thrombosis is rare [9]. Venous thrombosis may occur in a similar mechanism to arterial thrombosis but acquired protein S deficiency can also be a cause [10]. Vascular thrombosis mostly occurs with a latent period of two to three weeks, which signifies the time for the development of autoantibodies to natural anticoagulants, leading to a widespread thrombotic process [11-14].

The presence of neurological complications with active disease in this patient signifies that the latency period for the development of autoantibodies is much shorter with the lab findings of protein S deficiency providing a strong correlation. Early treatment with acyclovir has reduced the duration of illness but reduction of complications is not explained [15,16]. This patient was treated with intravenous acyclovir as per current recommendations for the treatment of complicated varicella [17]. There was a sustained improvement from the fourth day onwards.

Emergency physicians should have a high degree of suspicion for CVST in patients with active or recent varicella infection for appropriate therapy, as delayed diagnosis and treatment increases mortality. This patient was put on LMWH as there was no contraindication for anticoagulation combined with supportive measures and physical therapy as per current therapeutic measures used in clinical practice [18]. Concomitant intracranial hemorrhage related to CVST is not a contraindication for heparin therapy [18]. He was extubated by day four and had a complete recovery by day 12 of hospital admission and was discharged.

## Conclusions

In conclusion, this case highlights the importance of early recognition and prompt management of CVST in patients with active varicella infection. Neurological complications, such as CVST, can significantly impact outcomes, but with timely intervention using acyclovir, anticoagulation, and supportive care, recovery is possible. Emergency physicians must maintain a high suspicion for CVST in varicella patients to prevent delays in diagnosis and improve prognosis. This case underscores the effectiveness of current treatment strategies, including LMWH and physical therapy, in achieving favorable outcomes.
